# Moms in motion: weight loss intervention for postpartum mothers after gestational diabetes: a randomized controlled trial

**DOI:** 10.1186/s12884-021-03886-3

**Published:** 2021-06-29

**Authors:** Briana J. Stith, Samantha M. Buls, Sarah A. Keim, Stephen F. Thung, Mark A. Klebanoff, Mark B. Landon, Steven G. Gabbe, Kajal K. Gandhi, Reena Oza-Frank

**Affiliations:** 1 Center for Biobehavioral Health, The Research Institute at Nationwide Children’s Hospital, 700 Children’s Drive, Columbus, OH 43205 USA; 2 Department of Pediatrics, College of Medicine, The Ohio State University, 370 W. 9th Avenue, Columbus, OH 43210 USA; 3 Division of Epidemiology, College of Public Health, The Ohio State University, 250 Cunz Hall, 1841 Neil Avenue, Columbus, OH 43210 USA; 4 Department of Obstetrics and Gynecology, College of Medicine, The Ohio State University, 370 W. 9th Avenue, Columbus, OH 43210 USA; 5 Center for Perinatal Research, The Research Institute at Nationwide Children’s Hospital, 700 Children’s Drive, Columbus, OH 43205 USA; 6 Department of Pediatric Endocrinology, Nationwide Children’s Hospital, 700 Children’s Drive, Columbus, OH 43205 USA; 7 Ohio Department of Health, 246 N High Street, Columbus, OH 43215 USA

**Keywords:** Gestational diabetes mellitus, Clinical trial, Exercise, Physical activity, Lifestyle intervention, Prevention of type 2 diabetes mellitus, Weight loss

## Abstract

**Background:**

Up to 50 % of women with gestational diabetes mellitus (GDM) will receive a diagnosis of type 2 diabetes mellitus (T2DM) within a decade after pregnancy. While excess postpartum weight retention exacerbates T2DM risk, lifestyle changes and behavior modifications can promote healthy postpartum weight loss and contribute to T2DM prevention efforts. However, some women have difficulty prioritizing self-care during this life stage. Efficacious interventions that women can balance with motherhood to reduce T2DM risk remain a goal. The objective of the Moms in Motion study is to evaluate the efficacy of a simple, novel, activity-boosting intervention using ankle weights worn with daily activities during a 6-month postpartum intervention among women with GDM. We hypothesize that women randomized to the 6-month intensity-modifying intervention will (1) demonstrate greater weight loss and (2) greater improvement in body composition and biomarker profile versus controls.

**Methods:**

This study will be a parallel two-arm randomized controlled trial (*n* = 160). Women will be allocated 1:1 to an ankle weight intervention group or a standard-of-care control group. The intervention uses ankle weights (1.1 kg) worn on each ankle during routine daily activities (e.g., cleaning, childcare). Primary outcomes include pre- and post-assessments of weight from Visit 2 to Visit 3. Secondary outcomes include body composition, glycemia (2-h, 75 g oral glucose tolerance test), and fasting insulin. Exploratory outcomes include energy expenditure, diet, and psychosocial well-being.

**Discussion:**

Beyond the expected significance of this study in its direct health impacts from weight loss, it will contribute to exploring (1) the mechanism(s) by which the intervention is successful (mediating effects of energy expenditure and diet on weight loss) and (2) the effects of the intervention on body composition and biomarkers associated with insulin resistance and metabolic health. Additionally, we expect the findings to be meaningful regarding the intervention’s effectiveness on engaging women with GDM in the postpartum period to reduce T2DM risk.

**Trial registration:**

The ClinicalTrials.gov Identifier, is NCT03664089. The trial registration date is September 10, 2018. The trial sponsor is Dr. Sarah A. Keim.

## Background

The prevalence of gestational diabetes mellitus (GDM) has increased steadily in recent decades, affecting at least 7% of pregnant women in the United States (US) [1]. Up to 50% of women with GDM will develop type 2 diabetes mellitus (T2DM) within a decade after delivery [2]. Postpartum weight loss can reduce the risk of developing T2DM [3]. For instance, one study reported that each 10-pound increment of weight retained after pregnancy is associated with a 27% higher risk of T2DM [2], and a 2.5% decrease from baseline body weight is associated with a 60% reduction in T2DM incidence [4]. However, postpartum women have difficulty prioritizing self-care during this life stage [5]. Less than 33% of women with GDM undergo a postpartum oral glucose tolerance test, which is the first step in assessing postpartum T2DM risk and initiating prevention efforts [6].

Existing interventions for women with GDM require considerable time outside of already hectic schedules to change multiple behaviors. Arguably, the most effective weight loss interventions implement physical activity and dietary changes simultaneously. However, a sequential approach may be less overwhelming, place fewer demands on a person’s ability to change their behavior, require less effort [7], and be better suited for developing stronger habits long term [8]. Streamlined interventions may be even more feasible for women of low socio-economic status.

Three US-based studies have attempted to translate the Diabetes Prevention Program (DPP) for women with GDM [9–11] by modifying it to address lack of time as the strongest perceived barrier to behavior change [12, 13] (e.g., through telephone [9, 10], web [11], and mail [9]). In two of the studies, the intervention group retained less weight [10, 11], but this translated to only ~ 25% of participants, reflecting low levels of participation and adherence to the interventions. Two of these studies resulted in slight diet improvements [9, 11], and one resulted in increased self-reported physical activity [14]. Even if these interventions had shown more promising results, they were time- and resource-intensive, limiting scalability.

A recent pilot randomized controlled trial (RCT) tested the efficacy of a digital health support program to improve postpartum behaviors and glucose tolerance testing among women with GDM [15]. Participants received an activity monitor and motivational text messages integrated with their activity data. Although postpartum testing increased and the text messages yielded positive feedback from the participants, there was no significant difference between the control and intervention groups in dietary or physical activity goals achieved, and only 45% of participants completed the program. Another study examined weight loss and physical activity using a 3-month, web-based behavioral intervention amongst overweight or obese women with GDM [16]. The intervention consisted of a pedometer program and nutrition counseling with a sample of 28 Caucasian women and 3 women of Asian descent. Despite the small sample size, engaging postpartum women in behavior modification was challenging. Like previous studies, common barriers including lack of time, no childcare, and difficulty implementing lasting lifestyle changes reportedly affected all stages of the study and contributed to their difficulty recruiting and high attrition rate.

The lack of desired results and inconsistent success with previous studies is likely due to incomplete attention to aligning the type of intervention and intervention intensity to barriers postpartum women have to joining and adhering to lifestyle behavior change. It is evident that efficacious interventions to reduce T2DM risk that women can balance with motherhood remain a goal. Our intervention attempts to address this deficiency by adding more intensity to routine daily activities. New mothers are already engaged in an unavoidable lifestyle modification – they spend much of their time engaged in light-intensity physical activity such as cooking, cleaning, and childcare [17–21]. These same activities alone lower blood glucose [22]. We hypothesize that by increasing the intensity of these usual, daily activities, our intervention aims to maximize and increase energy expenditure to efficiently promote weight loss, an essential step towards reducing T2DM risk.

## Methods/design

### Specific aims

1. Compare the efficacy of an intervention focused on intensity modification during daily activities versus standard recommendations on measured weight loss (primary outcome).
2. Evaluate the impact of the intervention on body composition (i.e., % body fat, waist-hip ratio) and biomarkers associated with insulin resistance (i.e., glucose, insulin, HOMA, HbA1c, adiponectin, leptin) and metabolic health (i.e., lipids, blood pressure, hsCRP) (secondary outcomes).
3. Examine energy expenditure and diet as potential mediators to better understand the mechanisms behind greater weight loss in the intervention group (exploratory outcomes).

### Study design

The Moms in Motion study will be a parallel two-arm RCT to evaluate the efficacy of a simple, novel, physical activity-boosting intervention on postpartum weight loss among women with GDM.

### Setting

Women will be recruited from Ohio State University Wexner Medical Center Maternal-Fetal Medicine Diabetes in Pregnancy Clinics in Columbus, Ohio, USA. These clinics serve a large and diverse population, including both privately insured and publicly insured women referred from a large catchment area.

### Participants

Participants will be 160 women diagnosed with GDM during their current pregnancy. GDM is diagnosed with an oral glucose tolerance test using the Carpenter-Coustan criteria [23], typically between 24- and 28-weeks gestation. Testing may occur sooner if a patient has a higher risk of developing GDM.

This sample size was selected based on preliminary data and previous experience from our pilot study. We determined that a sample size of 160 women would be adequate and obtainable due to the number of eligible women we estimate to recruit and enroll, also considering an assumed 20% attrition rate. The recruitment clinics saw 419 unique patients with GDM in 2014. Based on preliminary data and previous experience, we estimate ≥ 80% of those women will be eligible (*n* = 335), of which > 90% will enroll resulting in a potential of *n* = 300/year. Our hypothesis is that women in the intervention will lose more weight than women in the control group. Sample size calculations were based on the difference in the pattern of weight means at study baseline and study endpoint (6-month intervention). Our preliminary data from our pilot studies indicate the intervention group mean weight loss from baseline to month 6 was 6.3 pounds vs. 3.9 pounds in the control group. Using linear mixed effect modeling to detect differences in mean weight change, we estimate an effect size (Cohen’s d) of 0.63. Using a more conservative d = 0.50, we estimate *n* = 64/group (*N* = 128) for 80% power. Assuming a 20% drop out rate, we need *n* = 80/group (*N* = 160), well within the estimated number of eligible women visiting the recruitment sites.

Eligible women will include those that provide written informed consent at 30–40 weeks’ gestation or 7–14 days postpartum, 18 years of age or older, English-speaking, plan to remain in the local area for the duration of the study and are physically capable of engaging in moderate physical activity. Women who are not English-speaking will be excluded because the resources for translating and validating standardized questionnaires for use in other languages are not currently available. Women who have a prior type 1 or type 2 diabetes diagnosis, are surrogate gestational carriers, are pregnant with multiples, deliver prior to 35 weeks’ gestation, have a pre-pregnancy BMI of less than 18.5 (underweight), or live more than 35 miles away from the Ohio State University Wexner Medical Center (Columbus, Ohio) will be ineligible.

**Fig. 1:**
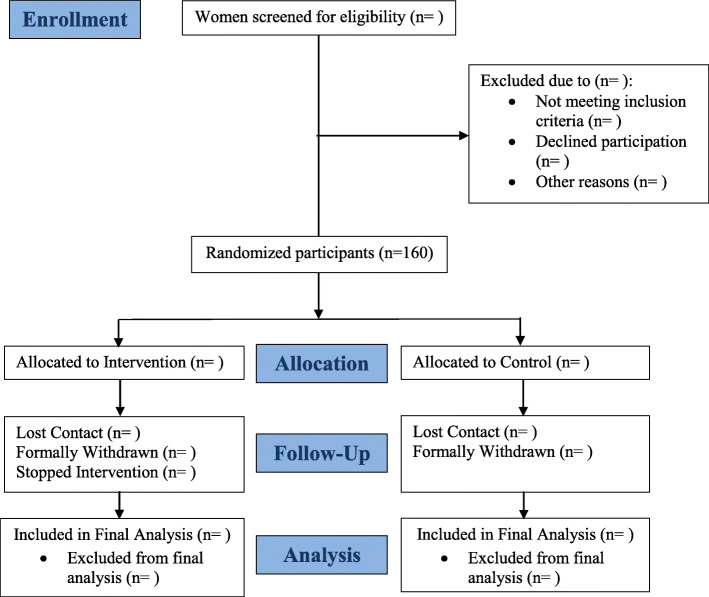
CONSORT Flow Diagram (Moms in Motion Study, Columbus, Ohio, USA)

Figure 1 provides the CONSORT flow diagram for the study, which is ongoing. It includes the total intended number for randomization. We do not have a total intended number for patients screened.

### Recruitment and informed consent

Clinic staff will distribute informational flyers to participants deemed eligible based on the inclusion and exclusion criteria and who attend the Diabetes in Pregnancy education class (first clinical point of contact after a GDM diagnosis). Study staff will then use the electronic medical record (EMR) and clinic schedule to approach eligible participants during their prenatal appointment to discuss the study and administer informed consent. Women who express interest will be eligible to consent at 30–40 weeks’ gestation prior to completing any study-related procedures. If they are not able to consent before delivery, they will have the opportunity during Visit 1, a home visit completed 7–14 days postpartum. Participants will receive a copy of Nationwide Children’s Hospital’s Notice of Privacy Practices at the point of consent, detailing their rights as a participant. All patients will have the potential to visit the clinic more than once after enrollment, allowing research staff to monitor subsequent visits of enrolled participants to remind them about the study and receive updates.

### Procedures: study visits and other contacts

There will be three study visits. Table 1 details the measures included in each visit. Table 2 shows the timeline for which these visits will be completed and the acceptable windows for these visits to be completed.

**Table 1:** Overview of Protocol Measures (Moms in Motion Study, Columbus, Ohio, USA)

Domains of Protocol Measures	Methodology	Data Source	Point of Data Collection					Data Collection Methods
			*Visit 1*	*Visit 2*	*Check-in Calls* (4)	*Visit 3*	*Follow-Up Call*	
Demographics and Medical Health Measures	Contact and sociodemographic factors (age, ethnicity, race, birthplace (country), average monthly income, government food assistance, insurance, parity, relationship status)	Surveys	X					REDCap software
	Medical History, Current Health, and Health Behavior		X	X	X (4)	X	X	REDCap software
	Delivery Outcomes		X					REDCap software
	New pregnancy		X	X	X (4)	X	X	REDCap software
Body Composition and Physical Activity Measures	Weight (kg)	In-person exam with study staff	X	X		X		Tanita TBF-310GS Body Composition Analyzer
	Percent body fat (%)		X	X		X		Tanita TBF-310GS Body Composition Analyzer
	Waist-hip circumference and ratio		X	X		X		Anthropometry performed by trained study staff
	Infant health (weight and length)			X		X		Anthropometry performed by trained study staff; infant scale and infantometer
	Energy expenditure and physical activity	ActiGraph wGT3X-BT device	X	X		X		ActiLife/Centrepoint Sync software
Glycemia and Associated Biomarkers of Insulin Resistance Measures	Glucose (mg/dl)	In-person exam with Clinical Research Services		X		X		Blood test
	Oral Glucose Tolerance Test			X		X		Blood test
	Insulin (pmol/l)			X		X		Blood test
	HOMA			X		X		Blood test
	Hemoglobin A1c (%)			X		X		Blood test
	Total cholesterol (mg/dl)			X		X		Blood test
	Triglycerides (mg/dl)			X		X		Blood test
	HDL-cholesterol (mg/dl)			X		X		Blood test
	LDL-cholesterol (mg/dl)			X		X		Blood test
	Systolic blood pressure (mmHg)			X		X		Vital sign
	Diastolic blood pressure (mmHg)			X		X		Vital sign
	Adiponectin (μg/ml)			X		X		Blood test
	Leptin (ng/ml)			X		X		Blood test
	hsCRP (mg/dl)			X		X		Blood test
Psychosocial Outcome Measures	Health Promoting Lifestyle Profile II	Surveys	X	X	X (1)	X	X	REDCap software
	Motivation/Confidence for Behavior Change		X	X	X (1)	X	X	REDCap software
	Body Shape Questionnaire		X	X	X (1)	X		REDCap software
	Diet History Questionnaire III			X		X		Web-based survey tool
	Breast/Infant Feeding		X	X	X (4)	X	X	REDCap software
	Pittsburgh Sleep Quality Index		X	X	X (1)	X	X	REDCap software
	Depression (CES-D)		X	X	X (1)	X		REDCap software
	Perceived Stress (PSS-10)		X	X	X (1)	X		REDCap software
Process Outcome Measures	Intervention acceptability, study satisfaction, suggestions for improvements	Surveys			X (1)	X	X	REDCap software

Table 1 outlines the protocol measures, methodology, data sources, points of data collection, and data collection methods that will be utilized in this study

**Table 2:** Visit Schedule (Moms in Motion Study, Columbus, Ohio, USA)

Study Visit	Days Postpartum	Days Post-Randomization	Acceptable Window
Visit 1: Home Visit	7–14 days	Not applicable	7–14 days postpartum
Visit 2: First Clinic Visit	30 days	0 days	25–35 days postpartum
Visit 3: Final Clinic Visit	220 days	190 days	190–210 days post-randomization

#### Visit 1 and run-in period

The first study visit (Visit 1) will take place in the participant’s home 7–14 days postpartum. Study staff will collect demographic information and data regarding current health (e.g., delivery outcomes, medical history). They will also collect initial measures of health behavior, lifestyle habits [24], motivation and confidence for behavior change [25, 26], perceptions of body shape [27], infant feeding practices, sleep quality [28], mental health and depressive symptoms [29], and perceived stress [30]. Study staff will measure the participant’s weight and body composition using the Tanita TBF-310GS Body Composition Analyzer (previous model), and they will measure hip circumference and waist circumference using metric tape. Before conducting measurements, study staff will complete anthropometric measurement training to ensure consistency and accuracy.

During the visit, the participant will receive an ActiGraph wGT3X-BT accelerometer to wear for all waking hours during a 7-day run-in period after Visit 1. Women who wear the accelerometer for more than 49 h over the 7-day run-in period will be invited to continue in the study; others will be dismissed.

#### Visit 2

Visit 2 will occur 25–35 days postpartum in clinical research facilities at Nationwide Children’s Hospital in Columbus, Ohio (Table 2). Body measurements, including weight, will be repeated. A registered nurse will collect biomarker measurements of insulin resistance via venipuncture, including a fasting 2-h, 75 g oral glucose tolerance test (OGTT); insulin; hemoglobin A1c; lipid panel; adiponectin; leptin; and high sensitivity C-reactive protein. A point-of-care glucose test will be performed at the beginning of each visit and will have a threshold of 250 mg/dL; if a participant’s fasting glucose is above the threshold, the principal investigator and registered nurses will investigate the situation to determine whether the participant should be withdrawn or rescheduled. The body composition and biomarker measurements completed at this visit will serve as the baseline measures. All Visit 1 questionnaires will be completed for a second time with the addition of the Diet History Questionnaire III (DHQ III). All participants will wear the ActiGraph wGT3X-BT accelerometer for 21 days after Visit 2 to monitor activity and energy expenditure. Study staff will share all blood test results with the participant and their OB/GYN or primary care provider via a letter sent in the mail stating that the results were collected for a research study and that the research staff will not provide follow-up care. Study staff will encourage participants to follow-up with their providers to discuss their results.

#### Randomization and blinding

Randomization will occur at the end of Visit 2. Research staff determine a participant’s eligibility before Visit 2. A pseudorandom number generator in the statistical software package SAS will be used to implement randomization, stratified by study clinic and BMI group with randomly varying block sizes. MAK will maintain the randomization sequence that will be unavailable to other research study members. Women will be randomized 1:1 to either the intervention or the control condition, stratified by clinic site and BMI category (18.5–24.9 or ≥ 25). The stratifying variables will be entered into REDCap by the research staff, and the concealed randomization algorithm will be programmed in REDCap and used by research staff to randomize women sequentially. Blinding of study investigators, staff, and women will not be possible with this intervention.

There will be five data collection calls. Table 3 shows the timeline for which these calls will be completed and the acceptable windows for these calls to be completed. Table 1 details the measures included in each data collection call.

**Table 3:** Data Collection Call Schedule (Moms in Motion Study, Columbus, Ohio, USA)

Data Collection Call	Days Postpartum	Days Post-Randomization
Check-in Call #1: End of Month 2	90 days	60–65 days
Check-in Call #2: End of Month 3	120 days	90–95 days
Check-in Call #3: End of Month 4	150 days	120–125 days
Check-in Call #4: End of Month 5	180 days	150–155 days
Follow-Up Phone Call	360 days	330–340 days

#### Phone call data collection

Women will participate in a series of data collection calls and surveys via a REDCap email link at 90, 120, 150, and 180 days postpartum (Table 3). During the phone calls, study staff will answer all study-related questions and document any health changes that may have occurred since their last encounter. Staff will encourage women to continue participating (i.e., continue wearing ankle weights, completing ankle weight journal logs, and completing surveys) by reinforcing the value of their participation.

#### Visit 3

Visit 3 will occur 190–210 days post-randomization in clinical research facilities at Nationwide Children’s Hospital (Table 2). It will repeat all measures from Visit 2. The body composition and biomarker measurements at this visit will serve as the final measures. As with Visit 2, study staff will share all blood test results with the participant and their OB/GYN or primary care provider via a letter sent in the mail stating that the results were collected for a research study and that the research staff will not provide follow-up care. Study staff will encourage participants to follow-up with their providers to discuss their results.

#### Final phone contact (follow-up phone call)

Women will participate in a final data collection call and surveys via a REDCap email link 360 days postpartum (Table 3). During the phone call, the participant will complete questionnaires, and study staff will collect data on their satisfaction with the study, intervention acceptability, and suggestions for improvements.

### Description of the intervention

Women assigned to the intervention will receive the standard recommendation to engage in 150 min of physical activity per week from their obstetric care provider. They will also receive a pair of ankle weights (2.5 pounds [1.1 kg] per ankle) after randomization during Visit 2 to wear until Visit 3. Study staff will instruct women to wear the ankle weights for 2 h every day during routine daily activities (i.e., childcare, household chores) and document the date, time, and activities completed in a study journal for the duration of the intervention. Women will be reminded and encouraged to send pictures of their study journal via email or text message. Study staff will send reminders as encouragement and appreciation weekly when they send in their journals.

Women assigned to the control arm will receive the standard recommendation from their obstetric care provider to engage in 150 min of physical activity per week. They will not receive a pair of ankle weights to wear after randomization, and they will not complete a study journal. This was selected as the control condition because it is the current standard of care.

### Primary outcome

#### Weight loss

The Tanita TBF-310GS Body Composition Analyzer will measure weight. Women will be weighed wearing light clothing and no shoes nor socks. We will account for 1 kg of clothing weight for every participant during each measurement. Postpartum weight loss will be defined as weight change between Visits 2 and 3.

### Secondary outcomes

#### Body composition

Height will be abstracted from the EMR before Visit 1 from the vitals section, which may be a measured or self-reported height. The Tanita will measure percent body fat, and trained study staff will measure waist and hip circumference using a metric tape. Waist circumference (cm) will be assessed at the middle point between the ribs and the iliac crest, with the participant in a standing position. Hip circumference (cm) will be measured at the widest circumference of the buttocks. Change in body composition measurements such as body fat percentage, BMI, and waist-hip ratio will be defined as the measurement change between Visits 2 and 3. The infant’s length and weight will be measured at Visits 2 and 3 using a calibrated infantometer and an infant scale.

#### Glycemia and associated biomarkers

The clinical laboratory at Nationwide Children’s Hospital will analyze all blood samples collected, except for adiponectin and leptin which will be sent to a partner laboratory. Insulin sensitivity and β-cell function will be measured using the Matsuda index94 and HOMA-IR.

### Potential mediators

#### Energy expenditure and physical activity

All women will receive an ActiGraph wGT3X-BT accelerometer to monitor their daily activity and energy expenditure. Using a belt clip, women will wear the ActiGraph on their waist for 21 days after Visit 2 and for 21 days before Visit 3. All devices will be calibrated based on the participant’s anthropometric data and configured for recording. Study staff will encourage women to sync their device daily to a phone application via Bluetooth technology. The application will upload data to a software system called CentrePoint, and only study staff will see the participant’s personal online profile in that system to allow for regular monitoring of compliance. For participants without access to a smartphone or Bluetooth technology, study staff will manually upload their data to hospital computers upon arrival at Visits 2 and 3. Women will receive customized reminder communications asking them to wear their ActiGraph upon request or if study staff observe that the participant is not wearing the device as often as desired. All participants will be blinded, such that no physical activity data can be viewed.

#### Diet

Participants will complete the Diet History Questionnaire III (DHQ III), a web-based food frequency questionnaire based on a collection of national 24-h dietary recall data from the National Health and Nutrition Examination Surveys (NHANES) conducted from 2007 to 2014.

#### Psychosocial assessments

Participants will complete surveys to assess changes in healthy lifestyle habits [24], sleep quality [28], motivation for behavior change [25, 26], perceptions of body shape [27], infant feeding practices, mental health and depressive symptoms [31], and perceived stress [30].

### Analysis

The study will adhere to CONSORT statement guidelines using intent-to-treat as the primary approach for analysis. All individuals will be kept in the group to which they were randomized, regardless of protocol violations or dropouts. Only the pre-specified covariates of baseline outcome variable, race, pre-pregnancy BMI category, GDM severity, parity, clinic location, and education will be included as covariates in the primary and secondary analyses unless imbalances are detected across arms in baseline variables [32]. Balance between the treatment groups will be tested by informally comparing baseline variables between study arms. Outliers will be checked and kept in the final analysis. Outcome variables will also be examined over time at the group and the individual level graphically as well as via descriptive statistics stratified on time and group. No interim analyses or stopping guidelines are planned regarding data monitoring.

Furthermore, bivariate relationships between outcome variables and potential covariates will be examined to determine if substantial deviations from linearity exist. We will make an effort to minimize dropouts and missing data, but if substantial missing data is realized, maximum likelihood estimation will be employed. Although not expected, if substantial non-compliance is realized, modern methods for handling missing data will be utilized [33, 34]. We will continue to collect all outcome data from participants who discontinue the intervention as long as the participant does not formally withdraw and is not lost to follow-up.

Analyses for the primary and secondary outcomes will involve a mixed effects model for repeated measures, similar to ANCOVA but based on maximum likelihood [35]. The same approach, statistical models, and time points (i.e., Visit 2 and 3) used for our primary hypothesis will be used for our secondary hypotheses, with each body composition and biomarker outcome in separate models instead of weight loss. For the Exploratory Aim, we will employ a longitudinal mediation model with multiple mediators. We will use Mplus 7.31 with bootstrapping procedures to statistically test mediators. We will follow the guidelines for testing longitudinal mediation within Cole and Maxwell and MacKinnon [36, 37]. These tests are important for clinical applicability of the intervention by identifying the most salient mechanisms of change that ultimately lead to better outcomes in our target population. Multiple publications will be submitted to peer-reviewed journals to disseminate study results.

### Participant safety and monitoring and data management

Human subjects review and approval are under the Nationwide Children’s Hospital’s Institutional Review Board (IRB) with a reliance agreement from the Ohio State University Medical Center’s IRB. The IRB has deemed the study “minimal risk.” Experienced research nursing staff will perform biospecimen collections and monitor patients during oral glucose tolerance tests. Study staff will assess adverse events at each study visit and during each data collection call via online surveys using REDCap software. The principal investigator and study doctor will review the adverse events monthly unless suspected to be Serious Adverse Events, and they will determine what action will be taken due to the adverse event regarding the study intervention. The study investigators will inform the IRB of all adverse events and will report all serious and related adverse events on an expedited timeframe per IRB guidelines.

The study will follow all necessary measures to assure the participant data stored in the database are protected and secure from unauthorized access. Only team members who are approved by the IRB to have access to the data will have access. Data use agreements with external collaborators may be possible as long as they are within the scope of the informed consent form. There is no planned auditing from the funder or other outside entity. Internal audits may be conducted on an unannounced basis.

## Discussion

The Moms in Motion study is innovative in its emphasis on simplicity to accelerate health improvement during a time when attention to self-health is not easily attainable. All new mothers experience physical and lifestyle changes and unavoidable time constraints postpartum, making it challenging to initiate sustainable weight loss efforts, which are essential for women with GDM to prevent/delay a T2DM diagnosis. The occurrence of GDM and high postpartum weight retention [1] continually contribute to the increasing number of women with T2DM. This study was designed to test an intervention to reduce T2DM risk among women who had GDM by considering the postpartum demands new mothers experience.

By testing an intervention that is sensitive to the postpartum demands new mothers experience, requiring minimal time and effort, we can shift current clinical paradigms to facilitate early weight loss and reduce T2DM risk among women with GDM. Beyond the expectation of this study to be significant in its direct health impacts from weight loss, it will be significant in exploring (1) the mechanism(s) by which the intervention is successful (mediating effects of energy expenditure and/or diet on weight loss) and (2) the effects of the intervention on body composition and biomarkers associated with insulin resistance and metabolic health.

Although this study is ongoing, we will expect the findings to be meaningful regarding the effectiveness of the intervention and how to engage women with GDM in the postpartum period to reduce T2DM risk. A limitation of this study is that we lack the resources to translate and validate questionnaires and other materials into other languages, so we must exclude women who do not speak English, which may reduce the generalizability of the findings. Also, participants will be followed to 12 months’ postpartum, but current resources do not permit longer-term follow-up. Future directions could incorporate following the participants over an extended period, beyond the duration of this study, to observe continued or sustained weight loss. Future research could also involve conducting a similar intervention in other settings or with larger samples adequately powered to robustly examine mechanisms.

## Data Availability

Not applicable.

## Abbreviations

GDM: Gestational diabetes mellitus
T2DM: Type 2 diabetes mellitus
OGTT: Oral glucose tolerance test
DPP: Diabetes Prevention Program
RCT: Randomized controlled trial
PI: Principal investigator
IRB: Institutional Review Board
